# Accuracy of the new radiographic sign of fecal loading in the cecum for differential diagnosis of acute appendicitis in comparison with other inflammatory diseases of right abdomen: a prospective study

**Published:** 2012-03-05

**Authors:** A Petroianu, LR Alberti

**Affiliations:** * Federal University of Minas Gerais, Belo Horizonte, Brazil; ** Santa Casa de Belo Horizonte and Federal University of Minas Gerais, Belo Horizonte, Brazil

**Keywords:** Appendicitis, Acute abdomen, Radiography, Cecum, Fecal loading

## Abstract

**Rationale: **To assess the importance of the new radiographic sign of faecal loading in the cecum for the diagnosis of acute appendicitis, in comparison with other inflammatory diseases, and to verify the maintenance of this radiographic sign after surgical treatment of appendicitis.

**Methods: **470 consecutive patients admitted to the hospital due to acute abdomen were prospectively studied: Group 1 [n=170] – diagnosed with acute appendicitis, subdivided into: Subgroup 1A – [n=100] – submitted to an abdominal radiographic study before surgical treatment, Subgroup 1B – [n=70] – patients who had plain abdominal X-rays done before the surgical procedure and also the following day; Group 2 [n=100] – right nephrolithiasis; Group 3 [n=100] – right acute inflammatory pelvic disease; Group 4 [n=100] – acute cholecystitis. The patients of Groups 2,3 and 4 were submitted to abdominal radiography during the pain episode.

**Results: **The sign of faecal loading in the cecum, characterized by hypo transparency interspersed with multiple small foci of hyper transparent images, was present in 97 patients of Subgroup 1A, in 68 patients of Subgroup 1B, in 19 patients of Group 2, in 12 patients of Group 3 and in 13 patients of Group 4. During the postoperative period the radiographic sign disappeared in 66 of the 68 cases that had presented with the sign. The sensitivity of the radiographic sign for acute appendicitis was 97.05% and its specificity was 85.33%. The positive predictive value for acute appendicitis was 78.94% and its negative predictive value was 98. 08%.

**Discussion: **The radiographic image of faecal loading in the cecum is associated with acute appendicitis and disappears after appendectomy. This sign is uncommon in other acute inflammatory diseases of the right side of the abdomen.

## Introduction

Among the manifestations of acute abdomen, pain in the right lower quadrant (RLQ) is probably one of the most challenging problems in medicine because ample possibilities of surgical and nonsurgical diseases must be taken into account [**1, 2, 3**]. The adequate management of these patients demands a precise diagnostic definition to establish the correct treatment. This decision requires data concerning the progression of the clinical picture associated with the physical examination, laboratory findings and imaging studies [**4, **].

Among the diseases that can cause an acute abdomen of the RLQ, acute appendicitis is the most common pathological condition. This disease is diagnosed on the basis of clinical examination, white blood cell count, abdominal ultrasound, CT scan and radiographic studies of the abdomen [**1**]. However, the less than perfect accuracy of these methods leads to an initial misdiagnosis rate of up to 20% in patients with pain in the right flank [**5**]. Misdiagnosis is more frequent in children, women and in the elderly. [**5, 6**].

Besides the episodes of acute appendicitis that are not diagnosed early, approximately 15% of all appendectomies result in the removal of apparently normal appendixes [**7-9**]. The association between acute appendicitis and skin color is another aspect subject to little investigation. Studies carried out in countries where the population is predominantly white-skinned revealed rates of appendicitis that reached 17%. On the other hand, in black populations of African countries where this disease is very rare, the incidence varied from 0.3 to 1% [**9-13**].

Delay in making a correct diagnosis can result in perforation, which is associated with high morbidity and even mortality, besides an increase in therapeutic costs [**14**]. False-positive diagnoses lead to unnecessary appendectomies, which also contributes to an increase in many undesirable effects [**15**].

In the presence of acute abdominal pain, plain abdominal radiographs are of great importance. Many radiographic signs have been related to appendicitis such as: appendicoliths (2 to 22% of the cases), gas in the appendix (< 2%), dilated small bowel loops with air-fluid levels confined to the lower right quadrant – sentinel loop – (15% a 55%), increase in soft-tissue density in the right lower quadrant (12% to 33%), loss or blurring of the properitoneal fat line (< 8%), deformity of the cecum contour (< 5%), separation of the cecal content from the right properitoneal fat (< 5%), abscesses, loss of the right psoas outline (1 to 8%), scoliosis concave to the right ( 1 to 14% of the patients) [**16**].

The purpose of this study was to assess the new radiographic sign characterized by fecal loading in the cecum in patients with acute appendicitis. Appendicitis was compared with other inflammatory diseases in order to verify its accuracy. The maintenance of this radiographic sign after surgical treatment was assessed as well.

## Patients and methods

This study complied with the recommendations of the Helsinki Declaration and the Resolution Nr. 196/96 of the Brazilian Ministry of Health concerning research involving human beings and was approved by the Research Ethical Committee of the Federal University of Minas Gerais. All the patients agreed to participate in the study by means of informed consent.

This prospective study was carried out on 470 consecutive patients of both sexes with abdominal pain localized in the right flank. Age, sex and skin color (white, brown and black) of all patients were acknowledged. 

Each patient received routine medical attention for acute abdomen that includes a complete physical examination, which included a gynecological exam when there were doubts concerning the cause of lower abdominal pain. Laboratory studies (complete blood count, urine and blood biochemical tests) and imaging studies (plain abdominal films, ultrasound imaging and abdominal CT scans) were carried out following the routine work-up when there were doubts concerning the diagnosis. It is important to stress that plain radiographs with an anteroposterior view of the abdomen were part of the complementary work-up in all cases, in accordance with the study protocol.

The patients were divided into four groups, according to their diseases:

Group 1 (n=170): patients of both sexes operated upon for acute appendicitis. The diagnosis was confirmed by the histological examination of the removed appendix. The histological criteria adopted to confirm the diagnosis of acute appendicitis was the presence of neutrophilic infiltrates in the muscularis of the appendix, besides other findings depending on the severity of the case [**17**]. These patients were subdivided into two subgroups:

- Subgroup 1A: (n = 100) – patients of both sexes (61 male and 39 female) ranging in age from 6 to 73 (31.33 ± 14.27) years old, of which 63 patients were white, 31 brown and 6 black. All of the patients underwent a radiographic study of the abdomen a few hours before surgical treatment.

- Subgroup 1B: (n = 70) – made up of patients of both sexes (40 men and 30 women) ranging in age from 5 days to 61 (18.71 ± 14.53) years old, of which 41 patients were white, 23 brown and 6 black. They all underwent two radiographic studies of the abdomen, one before the surgery and the other the day after the procedure.

Group 2 ([n = 100): patients of both sexes with calculi in the right urinary tract (88 cases of nephrolithiasis and 12 cases of ureterolithiasis). The group consisted of 40 men and 60 women, ages ranging from 4 to 84 (40.82 ± 14.48) years old, of which 54 patients were white, 37 brown and 9 black. Radiographs with an anteroposterior view of the abdomen, done supine and erect, were taken of all patients during the pain episode triggered by the calculi.

Group 3 (n = 100): patients with acute right gynecological affections ranging in age from 16 to 76 (32.60 ± 10.58) years old, of which 29 patients were white, 49 brown and 22 black. The conditions that motivated the hospital visit were acute hydrosalpinx in 63 patients, ruptured tubal pregnancy in 22 patients, rupture of an ovarian cyst in 14 patients and torsion of the right ovary in two cases. One 46-year-old patient presented with both hydrosalpinx and rupture of an ovarian cyst. Abdominal radiographs were taken of all patients during the pain episode that preceded treatment.

Group 4 (n = 100): patients of both sexes (30 men and 70 women) ranging in age from 17 to 90 (47.17 ± 18.28) years old, of which 35 patients were white, 54 brown and 11 blacks. They underwent surgery for acute cholecystitis and the diagnosis was confirmed during the procedure and by histological examination of the removed gallbladder. In eight patients there was cholangitis associated with cholecystitis. Plain radiographs with an anteroposterior view of the abdomen were obtained in all cases during the acute episode of pain that preceded surgical treatment.

The removed appendices were classified according to the macro- and microscopical morphologic stages: suppurated, fibrin purulent, gangrenous and perforated [**17**].

The patients were also classified according to time of pain: less than 12 hours, between 12 and 24 hours, between 24 and 72 hours or longer after the beginning of pain.

**Fig. 1:**
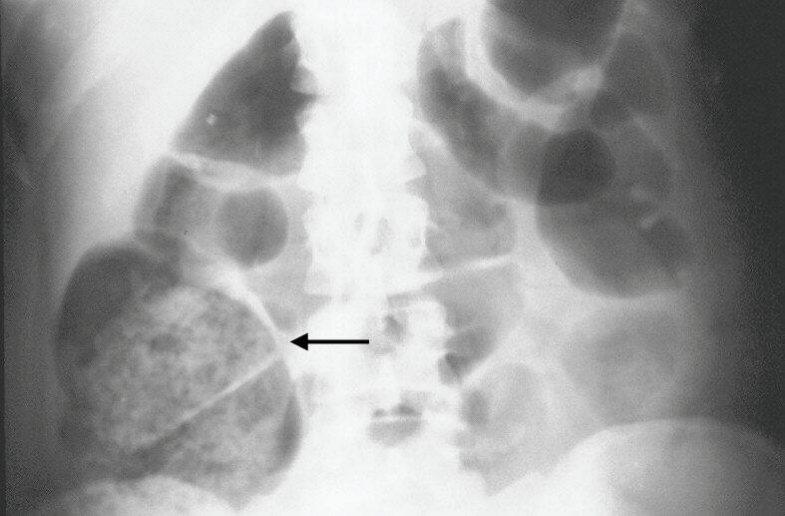
Plain abdominal radiographs of a patient with acute appendicitis. Observe the image of fecal loading in the cecum, which is distended (arrow).

The radiographic sign studied was the presence of an intraluminal image in the cecum, characterized by hypo transparency interspersed with multiple small foci of hyper transparent images (**Figure 1**).

This flake-like image, which is characteristic of fecal loading was eventually also seen in the ascending colon. All of the radiological procedures conducted in this study followed the routine recommendations for radiographic studies of the abdomen as to not expose the patients to atypical risks with respect to the proper work-up for acute abdominal pain [**18**]. Our findings were confirmed by a professor of radiology.

For statistical evaluation, the descriptive method of the mean and standard deviation of the mean of the patients' ages was employed. To compare the presence of the radiographic sign in the four groups, the chi-square test was applied. In Subgroup 1B the comparative analysis of the presence of the radiographic sign in the pre- and postoperative period was performed by the McNemar test for dichotomic variables in paired samples. The differences were considered significant for p values < 0.05 [**19**].

To assess the accuracy of the radiographic sign in detecting diseases, the sensitivity, specificity, positive predictive value (PPV) and negative predictive value (NPV) were calculated for all of the groups.

## Results

The presence of the radiographic sign in the four groups is shown in **Table 1**. There is a significant discrepancy when the presence of the radiographic sign of fecal loading in the cecum in Group 1 is compared to the presence of this sign in the other groups. There were no differences found among groups 2, 3 and 4 concerning the incidence of this sign. (**Table 1**)

**Table 1:** Image of fecal loading in the cecum on plain abdominal radiographs of patients with right acute abdominal pain

Disease		Radiographic sign
	[n]	Present	Absent
Acute appendicitis [Subgroup 1A]	100	97	3
[Subgroup 1B] *	70	68	2
Urinary calculi [Group 2]	100	19	81
Gynecologic affections [Group 3]	100	12	88
Biliary affections [Group 4]	100	13	87
* Different from groups 2, 3 and 4 (p < 0,0001).

The radiographic sign of fecal loading in the cecum was present in 165 patients diagnosed with acute appendicitis. The sensitivity of this sign for acute appendicitis was 97.05% and the specificity 85.33%. The positive predictive value of this sign for acute appendicitis was 78.94%, while the negative predictive value was 98.08% (**Table 2**).

**Table 2:** Comparison among the accuracy of statistical values for the sign of fecal loading in the cecum in patients with right acute abdominal pain

	SENSITIVITY	SPECIFICITY	PREDICTIVE VALUE
GROUP	(%)	(%)	POSITIVE (%)	NEGATIVE (%)
1	97.05 *	85.33 *	78.94 *	98.08 *
2	19.00	48.64	9.09	68.96
3	12.00	46.75	5.74	67.57
4	13.00	47.02	6.22	67.96
Group 1: acute appendicitis				
Group 2: urinary calculi				
Group 3: acute gynecologic inflammatory diseases				
Group 4: acute biliary inflammatory diseases				
* Different from groups 2, 3 and 4 (p < 0,0001).				

According to the morphology of the appendix in Subgroup 1A, 10 were suppurated, 60 fibrin purulent, 22 gangrenous and 8 were perforated. The radiographic sign was detected in all stages of the appendicitis. Of the three patients with and undetectable sign, two had fibrin purulent and one gangrenous acute appendicitis. There were no differences with respect to age, sex or skin color (p > 0.05). No difference was found in the incidence of perforated acute appendicitis related to gender (four men and four women) (p = 0.5081).

Considering the duration of the pain in patients of Subgroup 1A, eight were operated less than 12 hours after the beginning of symptoms, 58 between 12 and 24 hours and 25 patients between 24 and 72 hours. Nine patients underwent appendectomy 72 hours or longer after the beginning of pain. There was no association between the presence of the radiographic sign and the duration of preoperative pain (p > 0.05). However, seven of the nine patients with pain longer than 72 hours were females (p = 0.035)

The radiographic sign was present in the preoperative period in 68 patients and absent in two of the 70 patients of Subgroup 1B. In the postoperative period, the sign disappeared in 66 patients (p<0.001) yet persisted in one six-year-old brown patient and one thirty-eight-year-old white patient, both male.

The radiographic sign of fecal loading was present in all 18 children of Subgroup 1A. There were 49 pediatric patients in Group 2. With the exception of one eight-year-old boy, this sign was present in all children, including a 5-day-old premature newborn with perforated appendicitis.

Based on the morphologic classification of the appendix of Subgroup 1B, there were 13 suppurated, 39 fibrin purulent, 9 gangrenous and 9 perforated cases. Like in Group 1, the radiographic sign was present in all stages of the disease. There was no difference with respect to age, sex or skin color (p > 0.05).

Despite the greater number of males in Group 2 (40 males versus 30 females), there was a higher incidence of perforated acute appendicitis in women (eight out of the nine cases) (p = 0.002).

Considering the duration of pain in patients of Group 2, three had surgery less than 12 hours after the beginning of symptoms, 31 between 12 and 24 hours and 30 patients between 24 and 72 hours. Six patients underwent appendectomy 72 hours or longer after the beginning of pain. There was no association between the presence of the radiographic sign and the duration of preoperative pain (p > 0.05). However, five of the six patients with pain longer than 72 hours were females (p = 0.037).

The radiographic sign was found in only 19 cases in Group 2 (**Table 1**). These cases comprised 10 male and 9 female patients diagnosed with urinary calculi. There were 17 cases of nephrolithiasis and 2 cases of ureterolithiasis. The sensitivity of the radiographic sign for urinary calculi was 19.00% and the specificity 48.64%. The positive predictive value was 9.09% and the negative predictive value as 68.96% (**Table 2**).

The radiographic sign was found in only 12% of the patients of Group 3. Among these patients diagnosed with gynecologic diseases, 9 had hydrosalpinx, 2 had a ruptured tubal pregnancy and one had a ruptured ovarian cyst. Therefore, there was no correlation between the radiographic sign and the gynecologic disease (p=0.672). The sensitivity of the radiographic sign for gynecologic diseases was 12.00% and the specificity was 46.75%. The positive predictive value was 5.74% and the negative predictive value was 65.57% (**Table 2**).

The radiographic sign was present in only 13% of the patients of Group 4 (**Table 1**). Among these patients with biliary diseases, 3 were male (10% of the men of this group) and 10 were female (14.28% of the women of this group). One of these patients presented with acute cholangitis associated with acute cholecystitis. The sensitivity of the radiographic sign for biliary diseases was 13.00% and the specificity was 47.02%. The positive predictive value was 6.22% and the negative predictive value was 67.96% (**Table 2**).

## Discussion

The early diagnosis of acute appendicitis remains a challenge, principally among children, the elderly, debilitated or immune compromised patients and among women. This disease must be properly identified to avoid a delay in treatment and a consequent increase in morbidity [**14,20**]. The morbidity and mortality of acute appendicitis are due to the perforation of the appendix and the consequent formation of local, subphrenic, pelvic and multiple abscesses besides the risk of generalized peritonitis and a systemic inflammatory response [**21-24**].

Although the clinical features of this disease are well known, up to 25% of removed appendixes are apparently normal, especially among children and women [**21,25**]. It should be emphasized that one third of patients with acute appendicitis undergo surgical intervention with an uncertain preoperative diagnosis.

Although right urinary tract calculi do not cause peritoneal inflammation as occurs with appendicitis, acute cholecystitis and pelvic inflammatory disease, this group was added because it is an important differential diagnosis.

The incidence of perforation of the appendix was lower than that found in the literature. PENA et al. (2002) [**26**] submitted 1338 patients to an acute abdominal pain assessment protocol that comprised ultrasound imaging followed by an abdominal CT and reported that perforation had occurred in 15.5% of the cases of acute appendicitis. In this study, perforation occurred in 8% of the patients of Subgroup 1A and 9% of those of Subgroup 1B. These data indicate that perhaps the early diagnosis of acute appendicitis in this study prevented perforation. In this regard, the radiographic sign contributed to this favorable outcome because in many cases its presence was decisive to make the correct indication for surgery [**21,24,26**].

In spite of a larger number of men with appendicitis in this study, there was a higher incidence of perforation among women. This data conforms to the literature that mentions a greater difficulty in diagnosing acute appendicitis in women. This occurs on account of abdominal pain in this sex being related to numerous causes including painful ovulations, ovarian, tubal and uterine diseases and urinary tract infections, which are more common in women than in men ,[**27,28**] . Because of this difficulty, there was probably a delay in making the correct diagnosis, therefore favoring the progression of the disease to perforation. However, with this radiographic sign that appears in the early stages of appendicitis, maybe this disease can be treated without delay.

BARNES et al. (1962) [**29**] reported that more than 50% of the patients over 60 years old with acute appendicitis presented with minimal symptoms and were thus classified as “silent appendicitis”. In this situation, the disease progresses and this explains the high incidence of perforation and generalized peritonitis in elderly patients with acute appendicitis. It is important to consider the high morbidity and mortality in these patients because of the delay in making a diagnosis and starting treatment. In this work, the sign of fecal loading in the cecum occurred in all patients over 40 years old, consequently making possible the correct treatment without delay. If the pathology of acute appendicitis in the elderly patient is different than that of the disease in younger patients, the radiographic sign is still present in both cases.

According to FENKINS & LEE PETER (1970) [**30**], there is no pathognomonic radiographic sign for the diagnosis of acute appendicitis. BRADY & CARROL (1957) assessed the association between acute appendicitis and the appendicolith in 24 of 74 cases of acute appendicitis. This reduced incidence became even more unimportant when this sign was found in other digestive and gynecological diseases and even in healthy individuals [**31**].

Another sign associated with acute appendicitis is dilated small bowel loops with air-fluid levels confined to the lower right quadrant – sentinel loop. However, ONCEL et al. [2003] [**28**], while studying 162 patients with acute appendicitis, found this sign in 34.7% of the cases, an incidence lower than the 36.8% found in patients with other diseases. Besides occurring in a high number of patients with acute appendicitis, the sign of fecal loading in the cecum is also uncommon in other inflammatory diseases, and so it is important for the diagnosis of acute appendicitis.

Many imaging studies have been proposed to increase the diagnostic accuracy of acute appendicitis such as: ultrasound, Doppler ultrasound, CT scans, magnetic resonance imaging and scintigraphy. However, none of these more sophisticated methods has a greater accuracy than the radiographic sign here described [**32-34**]. According to PINTO LEITE et al. (2005), abdominal CT is a well-established technique in the study of acute abdominal pain and has shown high sensitivity and specificity for diagnosing and differentiating appendicitis, providing an accurate diagnosis in the early stages of this disease [**35**]. The radiographic sign here described has a lower specificity (85,33 % versus 100%) but a comparable sensitivity (97,05 % versus 97 %) with CT for the diagnosis of acute appendicitis [**36-38**].

Although the radiographic sign described is not pathognomonic of acute appendicitis, it offers strong indications for the diagnosis of this disease, especially if associated with a physical examination and laboratory findings. Surgical manipulation and CT scans performed in some cases confirmed that the radiographic image was indeed fecal loading.

In this casuistic, there were no misdiagnoses of acute appendicitis. Different stages of inflammation were related to the same radiographic finding. Only five of 170 patients without the radiographic sign presented appendicitis. All of them were followed during longer period until the correct diagnosis was established and then they were operated. Other patients without the radiographic sign had different diagnosis and were submitted to specific treatments, particularized to each case. It is important to be pointed that no patient without the radiographic sign was excluded from this study or discharged from the hospital without a correct treatment. Careful evaluation of these patients found: painful ovulation, mesenteric lymphadenitis and urinary lithiasis. None of them underwent surgery and the cause of pain was resolved with specific treatment.

New studies are in progress to explain the presence and the physiopathology of this radiographic sign in acute appendicitis. One hypothesis to explain the fecal loading would be the presence of a localized ileus in the cecum caused by the inflammatory process occurring next to it. After entering the cecum, the ileal content would be retained and its water content absorbed. During this period of chylous stasis caused by the cecal motor dysfunction, the formation of stool visible on the radiography would occur.

## Conclusion

As it was shown in previous preliminary studies [**39-40**], the present work confirmed that in the presence of acute appendicitis an image of fecal loading in the cecum is identified in almost all of the patients, irrespective of age, sex or skin color. The frequency of this sign is higher than that found in other acute inflammatory conditions of the right side of the abdomen. The absence of this sign makes the diagnosis of acute appendicitis improbable and after the removal of the appendix this image usually disappears.
